# Association of Days Alive and at Home at Day 90 After Intensive Care Unit Admission With Long-term Survival and Functional Status Among Mechanically Ventilated Patients

**DOI:** 10.1001/jamanetworkopen.2023.3265

**Published:** 2023-03-16

**Authors:** Shaurya Taran, Benjamin Coiffard, Ella Huszti, Qixuan Li, Leslie Chu, Claire Thomas, Stacey Burns, Priscila Robles, Margaret S. Herridge, Ewan C. Goligher

**Affiliations:** 1 Department of Neurology, Massachusetts General Hospital, Harvard Medical School, Boston; 2 Interdepartmental Division of Critical Care Medicine, University of Toronto, Toronto, Ontario, Canada; 3 Department of Respiratory Medicine, Assistance Publique-Hopitaux de Marseille, Aix-Marseille University, Marseille, France; 4 Institute for Health Policy, Management, and Evaluation, University of Toronto, Toronto, Ontario, Canada; 5 Toronto General Hospital Research Institute, Toronto, Ontario, Canada; 6 Institute of Medical Science, University of Toronto, Toronto, Ontario, Canada; 7 Division of Respirology, Department of Medicine, University Health Network, Toronto, Ontario, Canada

## Abstract

**Importance:**

Many conventional end points in randomized clinical trials of interventions for critically ill patients do not account for patient-centered concerns such as time at home, physical function, and quality of life after critical illness.

**Objective:**

To establish whether days alive and at home at day 90 (DAAH_90_) is associated with long-term survival and functional outcomes in mechanically ventilated patients.

**Design, Setting, and Participants:**

The RECOVER prospective cohort study was conducted from February 2007 to March 2014, using data from 10 intensive care units (ICUs) in Canada. Patients were included in the baseline cohort if they were aged 16 years or older and underwent invasive mechanical ventilation for 7 or more days. The follow-up cohort analyzed here comprised RECOVER patients who were alive and had functional outcomes ascertained at 3, 6, and 12 months. Secondary data analysis occurred from July 2021 to August 2022.

**Exposures:**

Composite of survival and days alive and at home at day 90 after ICU admission (DAAH_90_).

**Main Outcomes and Measures:**

Functional outcomes at 3, 6, and 12 months were evaluated with the Functional Independence Measure (FIM), the 6-Minute Walk Test (6MWT), the Medical Research Council (MRC) Scale for Muscle Strength, and the 36-Item Short Form Health Survey physical component summary (SF-36 PCS). Mortality was evaluated at 1 year from ICU admission. Ordinal logistic regression was used to describe the association between DAAH_90_ tertiles and outcomes. Cox proportional hazards regression models were used to examine the independent association of DAAH_90_ tertiles with mortality.

**Results:**

The baseline cohort comprised 463 patients. Their median age was 58 years (IQR, 47-68 years), and 278 patients (60.0%) were men. In these patients, Charlson Comorbidity Index score, Acute Physiology and Chronic Health Evaluation II score, ICU intervention (eg, kidney replacement therapy or tracheostomy), and ICU length of stay were independently associated with lower DAAH_90_. The follow-up cohort comprised 292 patients. Their median age was 57 years (IQR, 46-65 years), and 169 patients (57.9%) were men. Among patients who survived to day 90, lower DAAH_90_ was associated with higher mortality at 1 year after ICU admission (tertile 1 vs tertile 3: adjusted hazard ratio [HR], 0.18 [95% CI, 0.07-0.43]; *P* < .001). At 3 months of follow-up, lower DAAH_90_ was independently associated with lower median scores on the FIM (tertile 1 vs tertile 3, 76 [IQR, 46.2-101] vs 121 [IQR, 112-124.2]; *P* = .04), 6MWT (tertile 1 vs tertile 3, 98 [IQR, 0-239] vs 402 [IQR, 300-494]; *P* < .001), MRC (tertile 1 vs tertile 3, 48 [IQR, 32-54] vs 58 [IQR, 51-60]; *P* < .001), and SF-36 PCS (tertile 1 vs tertile 3, 30 [IQR, 22-38] vs 37 [IQR, 31-47]; *P* = .001) measures. Among patients who survived to 12 months, being in tertile 3 vs tertile 1 for DAAH_90_ was associated with higher FIM score at 12 months (estimate, 22.4 [95% CI, 14.8-30.0]; *P* < .001), but this association was not present for ventilator-free days (estimate, 6.0 [95% CI, −2.2 to 14.1]; *P* = .15) or ICU-free days (estimate, 5.9 [95% CI, −2.1 to 13.8]; *P* = .15) at day 28.

**Conclusions and Relevance:**

In this study, lower DAAH_90_ was associated with greater long-term mortality risk and worse functional outcomes among patients who survived to day 90. These findings suggest that the DAAH_90_ end point reflects long-term functional status better than standard clinical end points in ICU studies and may serve as a patient-centered end point in future clinical trials.

## Key Points

**Question:**

Can days alive and at home at day 90 (DAAH_90_)—a composite of mortality and the number of days at home at day 90 after intensive care unit (ICU) admission—be used to capture the burden of acute hospitalization and long-term functional outcomes in patients who require at least 7 days of mechanical ventilation?

**Findings:**

In this cohort study of 463 patients receiving invasive ventilation across 10 medical or surgical ICUs in Canada, lower DAAH_90_ was associated with multiple markers of higher ICU intensity and with worse functional outcomes and higher mortality up to 1 year following ICU discharge.

**Meaning:**

The findings of this study suggest that DAAH_90_ reflects the burden of critical illness and captures the long-term consequences of critical illness for functional status, quality of life, and survival.

## Teaser

This cohort study examines the association of a composite measure of mortality and number of days at home at day 90 after intensive care unit admission with long-term functional outcomes and survival among mechanically ventilated patients.

## Introduction

Critical illness is associated with important long-term decrements in functional status and quality of life.^1,2^ For many patients, avoiding substantial functional impairment is an equal if not greater priority than survival.^2,3^ Accordingly, measurements used to characterize the outcome of critical illness would ideally reflect both survival and survivorship—the impact of critical illness on long-term functional status and quality of life among survivors.

One measure that may reflect a patient-centered evaluation of the outcome of critical illness is days alive and at home (DAAH); similar constructs include hospital-free days or days alive and out of the hospital.^4,5,6,7^ Patients highly value time spent at home.^8^ By contrast, the experience of being hospitalized or admitted to a long-term acute care hospital can impair personal well-being and quality of life, especially if it occurs at the expense of time at home.^9^ Days alive and at home combines survival with time spent at home into a single measure that can be evaluated over different time points (eg, 3, 6, and 12 months). Patients who die within that period are often (although not necessarily) assigned a value of 0. For survivors, DAAH reflects hospital length of stay (LOS), hospital readmission, and admission to posthospital facilities such as acute rehabilitation units and complex chronic nursing institutions. From a trial design perspective, DAAH offers a comparatively objective outcome measure that can be ascertained using patient self-reports, registries, or health administrative data, making it easier to record than many existing patient-centered measures of functional outcome.^10^ From a patient perspective, time spent outside the hospital may represent a more meaningful measure of intervention efficacy compared with other end points commonly used in critical care trials, such as ventilator-free days or organ failure–free days.

Days alive and at home and similar end points have been reported across a range of inpatient settings and disease contexts^4,9,11,12,13,14^ but, to our knowledge, not after critical illness. In this secondary analysis of the RECOVER cohort study,^15^ we aimed to establish whether DAAH reflects both survival and survivorship in terms of functional status and quality of life after critical illness. We hypothesized that patients with lower DAAH at day 90 (hereafter, DAAH_90_) would report greater impairments in functional status, lower health-related quality of life, and higher mortality in the year after intensive care unit (ICU) discharge. We further hypothesized that DAAH_90_ would be more strongly associated with long-term functional outcomes compared with other composite end points routinely used in studies of critical illness, including ventilator-free days and ICU-free days.

## Methods

### Data Sources

We conducted a secondary analysis of the RECOVER multicenter prospective cohort study of 2-year outcomes after critical illness. The RECOVER study was conducted at 10 university-affiliated, medical or surgical ICUs in Canada from February 2007 to March 2014.^15^ Patients aged 16 years or older receiving invasive mechanical ventilation for 7 or more days in an ICU and for any medical or surgical admitting diagnosis were eligible for inclusion. Procedures for enrollment and follow-up are detailed in the original report.^15^ Research ethics approval for the RECOVER study was obtained at each participating center, and written informed consent from the substitute decision-maker was obtained for patient enrollment. This report followed the Strengthening the Reporting of Observational Studies in Epidemiology (STROBE) reporting guideline for observational studies.

### Computation of DAAH

We calculated DAAH_90_ as the total number of days free of index hospitalization, subsequent hospital readmissions, or admission to a long-term care facility at day 90. The following equation was used:

DAAH_90_ = 90 – hospital LOS – hospital LOS_90_ – long-term care facility LOS_90_

where hospital LOS represents LOS for the index hospitalization, hospital LOS_90_ reflects total LOS for subsequent hospitalizations, and long-term care facility LOS_90_ quantifies total LOS in a long-term care facility. Data on hospital readmissions were collected using a combination of retrospective chart review and patient self-reports. Patients who died within 3 months or remained admitted to a hospital or long-term care facility for the full 3-month period were assigned a value of 0 days.

### Measurements

Functional outcomes were evaluated at 3, 6, and 12 months. Overall functional status was assessed using the Functional Independence Measure (FIM),^15^ a patient-centered tool that assesses 6 overall areas of function across motor and cognitive domains. The FIM determines the degree of functional dependence, burden of care in motor and cognitive domains, rehabilitation needs, and outcomes in both critically and noncritically ill patient populations.^15,16,17,18^ Exercise capacity was quantified with the 6-Minute Walk Test (6MWT),^19^ and muscle strength was evaluated using the Medical Research Council (MRC) Scale for Muscle Strength (range, 0-60; higher scores indicate normal muscle strength, lower scores indicate weakness and/or absent contractions).^20^ Quality of life assessed using the 36-Item Short Form Health Survey physical component summary (SF-36 PCS)^21^ was evaluated as a comparator with the FIM, 6MWT, and MRC measures.^22^ Posttraumatic stress disorder (PTSD) and depressive symptoms were examined using the Impact Event Scale–Revised (IES-R [range, 0-88; higher scores indicate greater levels of distress cused by traumatic events, lower scores indicate lower levels of distress])^23^ and the Beck Depression Inventory–II (BDI-II [range, 0-63; higher scores indicate greater depressive symptoms, lower scores indicate fewer depressive symptoms]),^24^ respectively. Mortality was recorded at 1 year. Phase I of the RECOVER trial identified 4 trajectories of varying functional outcome based on age and ICU LOS^15^; these disability risk groups were retained for this analysis.

### Statistical Analysis

We trichotomized DAAH_90_ into tertiles to account for skewness of the distribution and the high frequency of 0 values. Patients were compared across tertiles according to their baseline characteristics, comorbidities, illness severity, and ICU characteristics. Descriptive statistics are presented as the mean (SD) or median (IQR). The statistical significance of differences across DAAH_90_ tertiles was tested using 1-way analysis of variance or the Kruskal-Wallis test, as appropriate. The unadjusted associations of baseline characteristics (eg, age, sex, comorbidity as quantified with the Charlson Comorbidity Index [CCI] score, and illness severity quantified with Acute Physiology and Chronic Health Evaluation II [APACHE II] score) and variables reflecting the burden of critical illness (eg, duration of invasive mechanical ventilation, tracheostomy, receipt of kidney replacement therapy, and ICU LOS) with DAAH_90_ were evaluated with ordinal logistic regression.

For patients who were alive at day 90, survival at 1 year was compared among DAAH_90_ tertiles with Kaplan-Meier curves. Between-group differences were assessed for significance using the log-rank test. The independent association of DAAH_90_ with 1-year mortality in the follow-up cohort was evaluated with Cox proportional hazards regression models adjusted for age, sex, CCI score, and APACHE II score.

For patients who survived to 3 months and who had functional outcomes measured at 3, 6, and 12 months (the follow-up cohort), associations of DAAH_90_ tertiles with FIM, 6MWT, MRC, and SF-36 scores at 3, 6, and 12 months were quantified with linear regression. To further assess the incremental value of DAAH_90_ over existing composite metrics, associations of DAAH_90_, ventilator-free days at day 28 (VFD_28_), and ICU-free days at day 28 (ICU-FD_28_) were compared separately with FIM at 1 year. Models were fitted using tertiles of each metric, and correlations are reported using *R*^2^ values. These models were further adjusted for age, sex, APACHE II score, and CCI score.

Since long-term care LOS may be less burdensome than acute hospitalization and potentially even preferable to being at home in some contexts,^25,26^ these associations were also evaluated in a sensitivity analysis that modified DAAH_90_ computation to exclude long-term care facility LOS. All statistical tests were 2-tailed and were considered significant at *P* < .05. Statistical analyses were performed using R, version 4.0.5 (R Project for Statistical Computing). Data analysis for this follow-up study was conducted between July 2021 and August 2022.

## Results

### Cohort Characteristics and Outcomes

**Figure 1.**
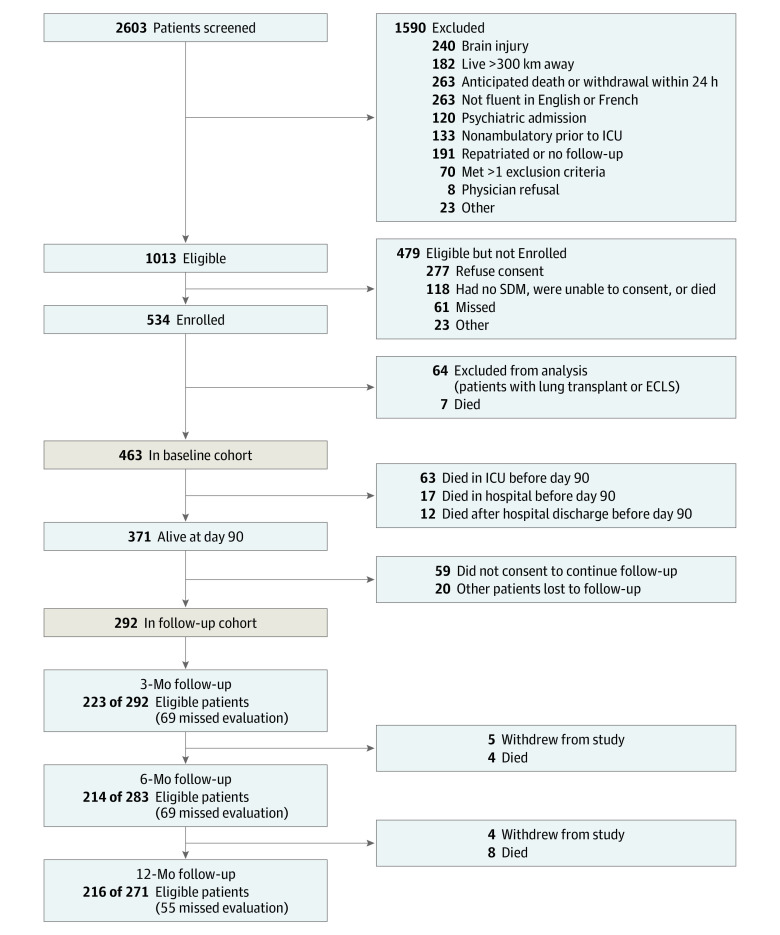
Participant Recruitment Flowchart ECLS indicates extracorporeal life support; ICU, intensive care unit; SDM, surrogate decision-maker.

A total of 463 patients were included in the baseline cohort (Figure 1). Their median age was 58 years (IQR, 47-68 years); 278 patients (60.0%) were men and 185 (40.0%) were women. Of these individuals, 292 were included in the follow-up cohort with functional outcomes measured at 3, 6, and 12 months (Figure 1). The median age in the follow-up cohort was 57 years (IQR, 46-65 years); 169 patients (57.9%) were men and 123 (42.1%) were women.

The distribution for DAAH_90_ is presented in eFigure 1 in Supplement 1. The median value was 11 days (IQR, 0-53 days). A total of 214 patients (46.2% of the overall cohort) had a DAAH_90_ of 0 days; 92 (19.9%) died and 109 (23.5%) remained in the hospital or other inpatient facility at 3 months. Thirteen patients (2.8%) were admitted to a long-term care facility. Removing long-term care LOS from the calculation of DAAH_90_ did not appreciably alter the distribution of days alive and at home (eFigure 1 in Supplement 1).

### Patient Characteristics and DAAH_90_ in the Baseline Cohort

Older age, higher CCI score, and higher APACHE II score were associated with lower DAAH_90_ (Table 1 and eFigure 2 in Supplement 1). Additionally, DAAH_90_ was not substantially different between men and women but varied considerably according to admission diagnosis (Table 1 and eFigure 2 in Supplement 1).

**Table 1.** Baseline and Critical Illness Characteristics According to Days Alive and at Home at Day 90 (DAAH_90_) in the Baseline Cohort^a^

Characteristic	DAAH_90_ (n = 463)			*P* value
	Tertile 1 (n = 214)	Tertile 2 (n = 102)	Tertile 3 (n = 147)	
DAAH_90_, median (range), d	0 (0-0)	27 (1-46)	63 (47-78)	NA
Age, median (IQR), y	61 (50-70)	59 (47-66)	54 (41-62)	<.001
Sex				
Men	134 (62.6)	64 (62.7)	80 (54.4)	.24
Women	80 (37.4)	38 (37.3)	67 (45.6)	
BMI, median (IQR)	26.7 (23.5-30.7)	27.7 (24.0-33.3)	28.0 (24.6-33.4)	.09
Education level				
Less than secondary	18 (8.4)	18 (17.6)	24 (16.3)	.001
Secondary or some postsecondary	33 (15.4)	27 (26.5)	39 (26.5)	
Postsecondary	163 (76.2)	57 (55.9)	84 (57.1)	
Prior employment				
Part-time or full-time	44 (48.4)	40 (50.6)	56 (50.5)	.11
Retired	27 (29.7)	23 (29.1)	20 (18.0)	
Disability	9 (9.9)	9 (11.4)	10 (9.0)	
Other	11 (12.1)	7 (8.9)	25 (22.5)	
Income, $				
<50 000	22 (24.2)	22 (27.8)	45 (40.5)	.11
50 000-70 000	10 (11.0)	11 (13.9)	11 (9.9)	
>70 000	26 (28.6)	20 (25.3)	32 (28.8)	
Unknown	33 (36.3)	26 (32.9)	23 (20.7)	
Charlson Comorbidity Index score, median (IQR)	2 (1-3)	1 (0-3)	1 (0-2)	<.001
Admission diagnosis category				
Cardiac	22 (10.3)	5 (4.9)	13 (8.8)	.08
Gastrointestinal	29 (13.6)	15 (14.7)	20 (13.6)	
Neurological	32 (15.0)	19 (18.6)	21 (14.3)	
Respiratory	60 (28.0)	28 (27.5)	51 (34.7)	
Sepsis	38 (17.8)	11 (10.8)	9 (6.1)	
Trauma	16 (7.5)	14 (13.7)	14 (9.5)	
Other	17 (7.9)	10 (9.8)	19 (12.9)	
Acute Physiology and Chronic Health Evaluation II score, median (IQR)	24 (18-28)	21 (17-26)	22 (15-28)	.03
Lowest Pao_2_/FiO_2_, median (IQR)	100 (66-142)	105 (74-173)	100 (74-144)	.23
Kidney replacement therapy	81 (37.9)	18 (17.6)	16 (10.9)	<.001
Tracheostomy	133 (62.1)	55 (53.9)	36 (24.5)	<.001
Duration of mechanical ventilation among ICU survivors, median (IQR), d	24 (14-36)	19 (13-27)	12 (9-17)	<.001
Ventilator-free days to day 28, median (IQR), d	4 (0-15)	9 (0-15)	17 (13-19)	<.001
ICU LOS among survivors, median (IQR), d	26 (18-45)	24 (16-33)	15 (11-19)	<.001
ICU-free days to day 28, median (IQR), d	0 (0-10)	4 (0-12)	13 (9-17)	<.001
Index hospitalization LOS, median (IQR), d	67 (33-111)	53 (43-68)	28 (22-37)	<.001
Days in hospital for subsequent hospital admissions, median (IQR)	21 (0-43)	19 (6-33)	0 (0-6)	<.001
Patients admitted in long-term care	12 (5.6)	1 (1.0)	0 (0.0)	.003
Functional Independence Measure score at day 7 after ICU discharge, median (IQR)	43 (30-57)	49 (31-66)	85 (55-107)	<.001
Total Medical Research Council Scale for Muscle Strength score, median (IQR)	38 (25-48)	46 (38-50)	52 (46-58)	<.001
Disability risk group^b^				
Young (<42 y), short LOS (<2 wk)	3 (1.4)	2 (2.0)	19 (12.9)	<.001
Mixed age (≥42 or ≤45 y), variable LOS (<2 or ≥2 wk)	53 (24.8)	36 (35.3)	70 (47.6)	
Older (46-66 y), long LOS (≥2 wk)	84 (39.3)	40 (39.2)	44 (29.9)	
Oldest (>66 y), long LOS (≥2 wk)	74 (34.6)	24 (23.5)	14 (9.5)	
Discharge disposition				
Acute or chronic care	32 (27.6)	8 (7.8)	18 (12.3)	<.001
Home	12 (10.3)	28 (27.5)	88 (60.3)	
Referring hospital	19 (16.4)	17 (16.7)	20 (13.7)	
Rehabilitation	53 (45.7)	49 (48.0)	20 (13.7)	
Died in ICU	61 (45.5)	0 (0.0)	0 (0.0)	<.001
Died in hospital	5 (2.3)	0 (0.0)	0 (0.0)	.05
Mortality at 1 year	126 (58.9)	8 (7.8)	6 (4.1)	<.001

Abbreviations: BMI, body mass index (calculated as weight in kilograms divided by height in meters squared); ICU, intensive care unit; LOS, length of stay; NA, not applicable; Pao_2_/FiO_2_, ratio of the partial pressure of arterial oxygen to fraction of inspired oxygen concentration.

^a^ Unless noted otherwise, data are expressed as No. (%) of patients.

^b^ Defined in the original RECOVER study.^15^

### Burden of Critical Illness and DAAH_90_ in the Baseline Cohort

In the baseline cohort, patients with a more prolonged duration of mechanical ventilation or ICU admission and those with lower VFD_28_ or ICU-FD_28_ had lower DAAH_90_ (Table 1 and eTable 1 in Supplement 1). Patients who required tracheostomy were more likely to be in a lower DAAH_90_ tertile (adjusted proportional odds ratio [OR], 0.31 [95% CI, 0.21-0.45]). Patients who required kidney replacement therapy were also more likely to be in a lower DAAH_90_ tertile (adjusted proportional OR, 0.23 [95% CI, 0.14-0.38]). The distribution of RECOVER disability risk groups and ICU LOS varied notably between DAAH_90_ tertiles in the original cohort (Table 1). Patients in higher risk groups had substantially lower DAAH_90_ (eFigure 3 in Supplement 1).

### DAAH_90_ and Long-term Functional Outcomes in the Follow-up Cohort

**Figure 2.**
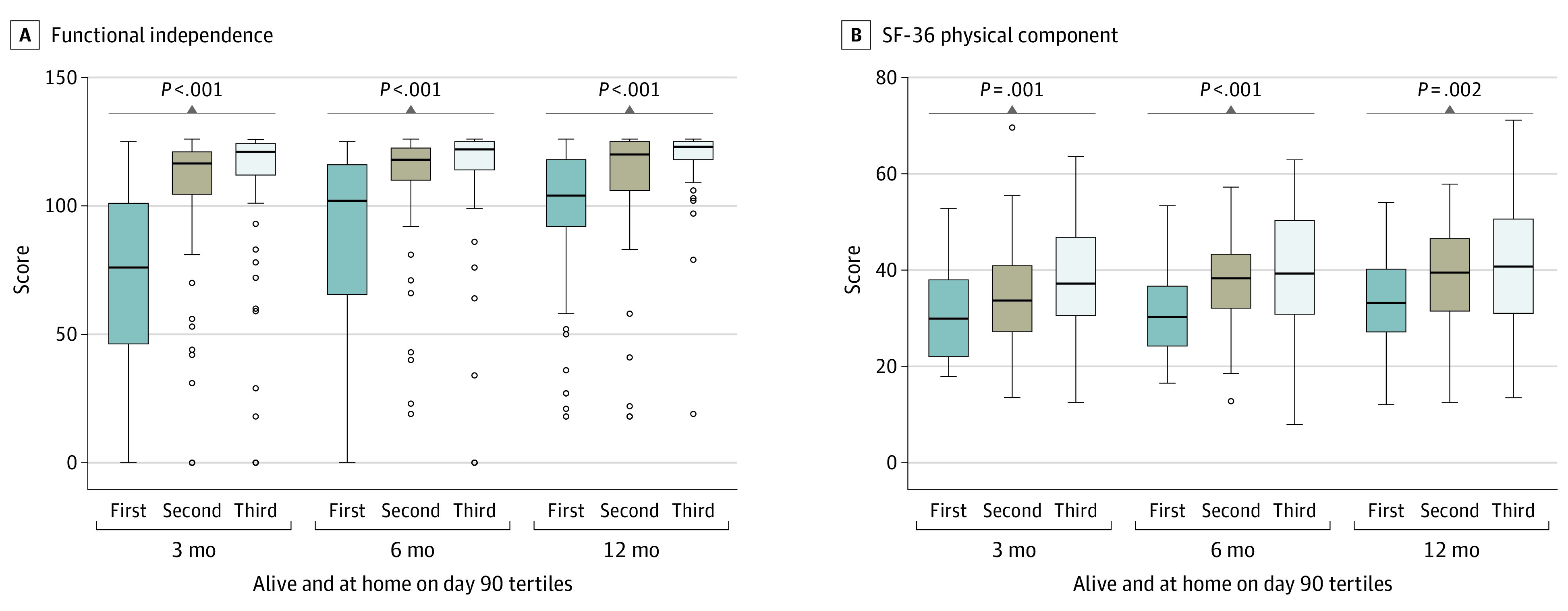
Association Between Tertiles of Days Alive and at Home at Day 90 (DAAH_90_) and Functional Outcomes at 3, 6, and 12 Months A and B, Data are derived from patients in the RECOVER follow-up cohort who survived to 3 months and who had functional outcomes measured at 3, 6, and 12 months using the Functional Independence Measure (FIM; A) and the 36-Item Short Form Health Survey physical component summary (SF-36 PCS; B). Higher FIM and SF-36 PCS scores indicate a greater level of functional independence. Lower values of DAAH_90_ are consistently associated with worse functional outcomes. Horizontal lines represent medians; the lower and upper bounds of the boxes represent the first and third quartiles of the data, respectively; whiskers encompass data extending to 1.5 times the IQR (derived as the difference between the first and third tertiles); and circles represent outliers.

Baseline and critical illness characteristics of the follow-up cohort are reported in eTable 2 in Supplement 1. In both unadjusted and adjusted analyses, lower DAAH_90_ was associated with lower FIM scores at 3, 6, and 12 months (Figure 2 and Table 2). Among patients who survived to 12 months, being in tertile 3 vs tertile 1 for DAAH_90_ was associated with higher FIM score (estimate, 22.4 [95% CI, 14.8-30.0]; *P* < .001), but this association was not present for VFD_28_ (estimate, 6.0 [95% CI, −2.2 to 14.1]; *P* = .15) or ICU-FD_28_ (estimate, 5.9 [95% CI, −2.1 to 13.8]; *P* = .15) (eTable 3 in Supplement 1). Lower tertiles of DAAH_90_ were also associated with lower 6MWT, MRC, and SF-36 scores at 3, 6, and 12 months (eg, at 3 months: 6MWT tertile 1 vs tertile 3, 98 [IQR, 0-239] vs 402 [IQR, 300-494], *P* < .001; MRC tertile 1 vs tertile 3, 48 [IQR, 32-54] vs 58 [IQR, 51-60], *P* < .001; SF-36 tertile 1 vs tertile 3, 30 [IQR, 22-38] vs 37 [IQR, 31-47], *P* = .001) (Figure 2 and Table 2). However, DAAH_90_ was not associated with IES-R or BDI-II scores at any of the time points measured (Table 2). In a sensitivity analysis in the follow-up cohort excluding long-term care LOS from DAAH_90_ calculation, the association of DAAH_90_ with functional outcomes persisted (eTable 4 in Supplement 1). In a sensitivity analysis partitioning DAAH_90_ into deciles, FIM, SF-36, and MRC scores progressively increased with increasing DAAH_90_, although the magnitude of increase varied across the range of deciles (eFigure 5 in Supplement 1).

**Table 2.** Functional Outcomes at 3, 6, and 12 Months According to Days Alive and at Home at Day 90 (DAAH_90_) for Patients Alive at 3 Months in the Follow-up Cohort

Outcome measure	DAAH_90_ (n = 292)^a^			*P* value	
	Tertile 1 (n = 98)	Tertile 2 (n = 97)	Tertile 3 (n = 97)	Unadjusted	Adjusted^b^
DAAH_90_, median (range), d	0 (0-1)	30 (2-52)	65 (53-78)	NA	NA
Functional Independence Measure score					
3 mo	76 (46.2-101)	116.5 (104.5-121)	121 (112-124.2)	<.001	.04
6 mo	102 (65.6-116)	118.00 (110-122.5)	122 (114-125)	<.001	.03
12 mo	104 (92-118)	120 (106-125)	123 (118-125)	<.001	<.001
6-Minute Walk Test score					
3 mo	98 (0-239)	337 (240-451)	402 (300-494)	<.001	<.001
6 mo	264 (101-354)	400 (272.2-491.2)	443 (296-516)	<.001	<.001
12 mo	325 (144-409)	408 (252-507)	449.5 (370-548)	<.001	<.001
Medical Research Council Scale for Muscle Strength score					
3 mo	48 (32-54)	53 (48-58)	58 (51-60)	<.001	<.001
6 mo	48 (44-58)	58 (48-60)	60 (56-60)	<.001	<.001
12 mo	55 (48-60)	60 (50-60)	60 (58-60)	<.001	<.001
36-Item Short Form Health Survey physical component summary score					
3 mo	30 (22-38)	34 (27-41)	37 (31-47)	.001	.001
6 mo	30 (24-37)	38 (32-43)	39 (31-50)	<.001	<.001
12 mo	33 (27-40)	40 (32-47)	41 (31-51)	.002	<.001
Impact Event Scale–Revised score					
3 mo	15 (3-26)	13 (4-27)	18 (5-31)	.58	.83
6 mo	12 (4-28)	13 (4-28)	15 (5-28)	.69	.56
12 mo	14 (8-27)	10 (2-23)	11 (4-27)	.12	.12
Beck Depression Inventory–II score					
3 mo	12 (7-18)	9 (6-16)	9 (6-17)	.37	.24
6 mo	11 (5-18)	8 (3-13)	10 (4-19)	.13	.05
12 mo	10 (6-18)	8 (4-14)	8 (2-17)	.28	.33
Mortality at 1 year	11 (11.2)	5 (5.2)	1 (1.0)	.009	.006

Abbreviation: NA, not applicable.

^a^ Unless noted otherwise, data are expressed as the median (IQR).

^b^ Multivariable models were adjusted for age, sex, Acute Physiology and Chronic Health Evaluation II score, and Charlson Comorbidity Index score.

### DAAH_90_ and 1-Year Mortality

**Figure 3.**
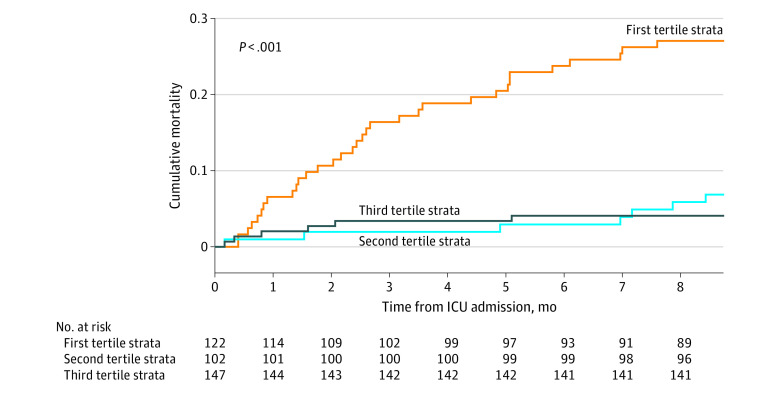
Cumulative Mortality Up to 12 Months After Intensive Care Unit (ICU) Discharge According to Tertiles of Days Alive and at Home at Day 90 Among Patients Alive at Day 90 Patients in the lowest tertile of days alive and at home at day 90 had a significantly (*P* < .001) higher risk of death at 1 year compared with patients in higher tertiles.

After restricting the unadjusted analysis to the 371 patients alive at day 90, lower DAAH_90_ was associated with a higher risk of death at 1 year (tertile 2 vs tertile 1: adjusted HR, 0.28 [95% CI, 0.12-0.64]; *P* = .012, log-rank test of difference; Figure 3). In the adjusted analysis, higher DAAH_90_ tertiles were associated with a lower risk of death at 1 year compared with the lowest DAAH_90_ tertile (eTable 5 in Supplement 1). In a sensitivity analysis of patients alive at day 90 that excluded long-term care facility LOS from the computation of DAAH_90_, lower DAAH_90_ was persistently associated with higher mortality (eFigure 4 in Supplement 1). Finally, in patients alive at day 90, 1-year mortality was highest in lower deciles of DAAH_90_ (eFigure 6 in Supplement 1).

## Discussion

In this secondary analysis of the RECOVER prospective cohort study of adults receiving mechanical ventilation for 7 or more days across 10 Canadian ICUs,^15^ we observed that DAAH_90_ was associated with multiple measures of functional status and quality of life in ICU survivors over the first year after critical illness and with mortality at 1 year. In addition, we observed that DAAH_90_ reflected the severity of critical illness as represented by multiple metrics of ICU intensity. In this study, other common metrics of ICU outcomes (VFD_28_ and ICU-FD_28_) were not associated with long-term functional status. These findings support the use of DAAH_90_ as a readily ascertained patient-centered outcome in future clinical studies and clinical trials involving critically ill patients.

The DAAH measure prioritizes time spent at home while offering information about mortality and length of hospitalization.^5,13,27^ Prior studies have explored DAAH as a measure of intervention efficacy across various hospitalized populations.^5,6,9^ To our knowledge, this is the first study to evaluate the role of DAAH_90_ in the critical care setting. The DAAH_90_ outcome measure is attractive in critical care for several reasons. First, DAAH_90_ prioritizes an outcome that most individuals would consider important (time spent at home). Many conventional end points in critical care research do not capture priorities of direct importance to patients and may be difficult for patients to comprehend.^28^ Mortality is convenient to measure and important to patients, but it may be superseded by other priorities that are more relevant to patients in certain contexts, such as avoiding pain or preserving quality of life.^2,29,30^ Organ failure–free days, ventilator-free days, and ICU-free days have similar shortcomings. Indeed, we observed that these end points were not associated with long-term functional status in ICU survivors. Because health is widely regarded as more than mere survival, outcomes such as DAAH_90_ may be useful for measuring what matters most to patients.^31^

Second, from a trial design perspective, DAAH_90_ can be easily computed from site-submitted data or health administrative registries without time-intensive adjudication.^14^ Critical care end points often require central event adjudication according to specific criteria (eg, classification of organ failures), potentially creating challenges in comparing outcomes across studies where adjudication criteria were different.^14^ Our results suggest that the use of DAAH_90_ overcomes this limitation because it incorporates data on outcomes that are objective, easy to obtain, and comparable across studies.

Third, use of DAAH_90_ as a common end point may enable comparisons across studies in which interventions serve different goals.^32^ Studies of therapeutic interventions frequently evaluate outcomes in terms of morbidity or mortality, whereas those evaluating palliative interventions often report outcomes in terms of health-related satisfaction or quality of life. Direct comparisons between these interventions may be challenging because the 2 outcomes evaluate different patient priorities. In this context, the use of DAAH_90_ across studies is likely to be appreciated by patients because it portrays the risk of undesirable outcomes (eg, death or longer inpatient stays) alongside a desirable outcome (eg, being at home), thus permitting a fairer comparison and selection of the more goal-concordant intervention.^32^

Clinicians and investigators seeking to use DAAH_90_ should consider the following additional points to ensure that the metric is appropriate for their intended uses. First, DAAH_90_ assumes that time spent at home is preferable to any time in the clinical setting, which is not always true. Some patients may find the home environment uncomfortable or improperly equipped to deal with their care needs and may prefer to receive care in a hospital.^32^ Second, composite outcomes such as DAAH_90_ assign equal weight to the components included in the calculation, and this weighting may not be precisely aligned with individual patient values.^28,33^ As with all composite outcomes, the individual components of DAAH_90_ should be reported. Some have also argued that it is not appropriate to automatically assign a value of 0 for all patients who die within the time period considered, since time at home before death may be of real value to patients.^32^ Third, the statistical gains that make composite measures such as DAAH_90_ appealing may disappear entirely if the intervention causes the components of the composite to change in different directions.^28^ A study evaluating a rapid discharge strategy from acute care settings could potentially decrease hospital LOS at the expense of increasing long-term care LOS; such an intervention might be ill suited for evaluation with DAAH_90_. Careful a priori consideration of how the proposed intervention might affect each component of the composite outcome is therefore required in order to maximize the statistical utility of this measure.

### Limitations

This study has some limitations. The original RECOVER cohort included patients who required mechanical ventilation for at least 7 days. The median duration of mechanical ventilation among survivors discharged from the ICU was 16 days, and the median hospital LOS for the overall cohort was 49 days.^15^ These durations are substantially longer than those from comparable cohort studies, in which the median duration of mechanical ventilation ranged from 3.0 to 4.1 days and hospital LOS was 18.9 to 22.5 days.^34,35^ Whether DAAH_90_ is a relevant outcome among nonventilated patients or those with shorter hospitalizations remains unknown. Future studies could aim to explore DAAH_90_ in more heterogeneous ICU cohorts. Our calculation of DAAH_90_ also assumes that any days not spent in a hospital or long-term care facility were spent at home. In reality, 5.2% of patients in the original cohort not discharged home or to long-term care were transferred to rehabilitation, assisted living, or chronic care.^15^ Obtaining data on discharges to these alternative institutions is not always possible and may limit the applicability of the described measure. Nevertheless, to offer the most realistic portrayal of a proposed intervention, it should be made clear to patients how much home time is truly contributed by time at home vs time spent in a nonacute inpatient facility.^36^ In addition, DAAH_90_ does not consider the impact of home time on patient caregivers, their ability to provide care outside the hospital, and the potential for caregiver burnout, which may limit the success of home discharge.^37,38^ Data on caregiver status were only collected for a portion of patients enrolled in the original RECOVER study.^15^ Finally, our findings suggest that DAAH_90_ does not adequately capture the long-term burden of mental illness following critical illness. We did not observe an association between DAAH_90_ and IES-R or BDI-II scores at any of the follow-up time points that are specific to PTSD and depression.

## Conclusions

In this cohort study, DAAH_90_ was associated with long-term functional status and survival after critical illness. Given its comparative ease of ascertainment, DAAH_90_ represents a patient-centered end point that may be useful in future clinical studies and clinical trials in critical care.

## Notes

Supplement 1. **eFigure 1.** Frequency Distributions for Days Alive and at Home at Day 90 (DAAH_90_) With or Without Including Length of Stay (LOS) in Long-term Care **eFigure 2.** Distribution of Days Alive and at Home at Day 90 (DAAH_90_) According to Baseline Characteristics of the Study Population **eTable 1.** Association of Measures of Burden of Critical Illness With Tertiles of Days Alive and at Home at Day 90 **eTable 2.** Baseline Characteristics of the 292 Patients in the Follow-up Cohort **eFigure 3.** Distribution of Days Alive and at Home at Day 90 (DAAH_90_) According to Disability Risk Groups Defined in the RECOVER Study **eTable 3.** Adjusted Association of Composite Metrics With Functional Independence Measure Score at 1 Year **eTable 4.** Sensitivity Analysis Removing Long-Term Care Length of Stay at Day 90 **eTable 5.** Cox Proportional Hazards Regression Model for 1-Year Mortality **eFigure 4.** Survival at 12 Months Among Survivors at Day 90 According to Tertiles of Days Alive and at Home at Day 90 Removing Long-Term Care Length of Stay **eFigure 5.** Association of Deciles of Days Alive and at Home at Day 90 (DAAH_90_) With Functional Outcomes at 3 Months **eFigure 6.** Mortality at 1 Year in Survivors at Day 90 According to Deciles of Days Alive and at Home at Day 90 (DAAH_90_)

Supplement 2.
Data Sharing Statement
