# Supplementary material for: Association of Days Alive and at Home at Day 90 After Intensive Care Unit Admission With Long-term Survival and Functional Status Among Mechanically Ventilated Patients
Source: JAMA Netw Open. 2023 Mar 16;6(3):e233265. doi: 10.1001/jamanetworkopen.2023.3265 (PMC10020882; doi:10.1001/jamanetworkopen.2023.3265)
Supplement: Supplement 1. — eFigure 1. Frequency Distributions for Days Alive and at Home at Day 90 (DAAH90) With or Without Including Length of Stay (LOS) in Long-term Care eFigure 2. Distribution of Days Alive and at Home at Day 90 (DAAH90) According to Baseline Characteristics of the Study Population eTable 1. Association of Measures of Burden of Critical Illness With Tertiles of Days Alive and at Home at Day 90 eTable 2. Baseline Characteristics of the 292 Patients in the Follow-up Cohort eFigure 3. Distribution of Days Alive and at Home at Day 90 (DAAH90) According to Disability Risk Groups Defined in the RECOVER Study eTable 3. Adjusted Association of Composite Metrics With Functional Independence Measure Score at 1 Year eTable 4. Sensitivity Analysis Removing Long-Term Care Length of Stay at Day 90 eTable 5. Cox Proportional Hazards Regression Model for 1-Year Mortality eFigure 4. Survival at 12 Months Among Survivors at Day 90 According to Tertiles of Days Alive and at Home at Day 90 Removing Long-Term Care Length of Stay eFigure 5. Association of Deciles of Days Alive and at Home at Day 90 (DAAH90) With Functional Outcomes at 3 Months eFigure 6. Mortality at 1 Year in Survivors at Day 90 According to Deciles of Days Alive and at Home at Day 90 (DAAH90) [file jamanetwopen-e233265-s001.pdf]

Supplementary Online Content

Taran S, Coiffard B, Huszti E, et al. Association of days alive and at home at day 90 after intensive care unit admission with long-term survival and functional status among mechanically ventilated patients. *JAMA Netw Open*. 2023;6(3):e233265. doi:10.1001/jamanetworkopen.2023.3265

eFigure 1. Frequency Distributions for Days Alive and at Home at Day 90 ($DAAH_{90}$) With or Without Including Length of Stay (LOS) in Long-term Care

eFigure 2. Distribution of Days Alive and at Home at Day 90 ($DAAH_{90}$) According to Baseline Characteristics of the Study Population

eTable 1. Association of Measures of Burden of Critical Illness With Tertiles of Days Alive and at Home at Day 90

eTable 2. Baseline Characteristics of the 292 Patients in the Follow-up Cohort

eFigure 3. Distribution of Days Alive and at Home at Day 90 ($DAAH_{90}$) According to Disability Risk Groups Defined in the RECOVER Study

eTable 3. Adjusted Association of Composite Metrics With Functional Independence Measure Score at 1 Year

eTable 4. Sensitivity Analysis Removing Long-Term Care Length of Stay at Day 90

eTable 5. Cox Proportional Hazards Regression Model for 1-Year Mortality

eFigure 4. Survival at 12 Months Among Survivors at Day 90 According to Tertiles of Days Alive and at Home at Day 90 Removing Long-Term Care Length of Stay

eFigure 5. Association of Deciles of Days Alive and at Home at Day 90 ($DAAH_{90}$) With Functional Outcomes at 3 Months

eFigure 6. Mortality at 1 Year in Survivors at Day 90 According to Deciles of Days Alive and at Home at Day 90 ($DAAH_{90}$)

This supplementary material has been provided by the authors to give readers additional information about their work.

© 2023 Taran S et al. *JAMA Network Open*.

eFigure 1. Frequency Distributions for Days Alive and at Home at Day 90 ($DAAH_{90}$) With or Without Including Length of Stay (LOS) in Long-term Care

$DAAH_{90}$ including long term care LOS

$DAAH_{90}$ excluding long term care LOS

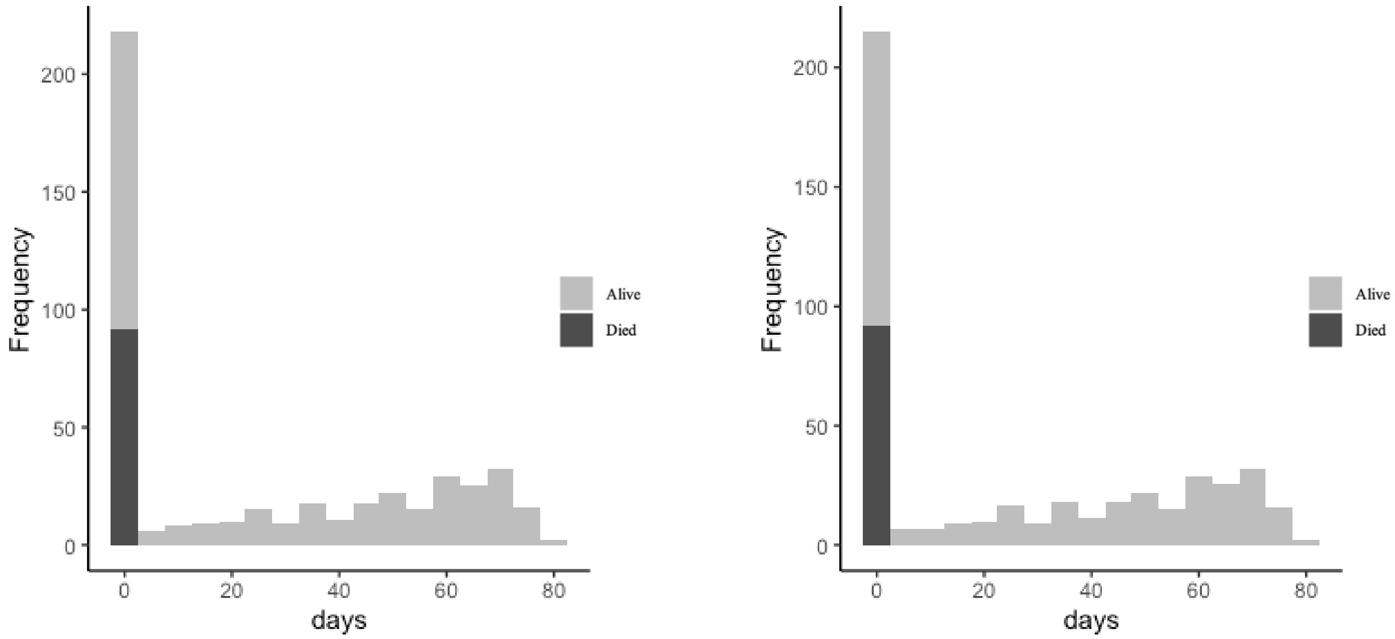

© 2023 Taran S et al. *JAMA Network Open*.

eFigure 2. Distribution of Days Alive and at Home at Day 90 ($DAAH_{90}$) According to Baseline Characteristics of the Study Population

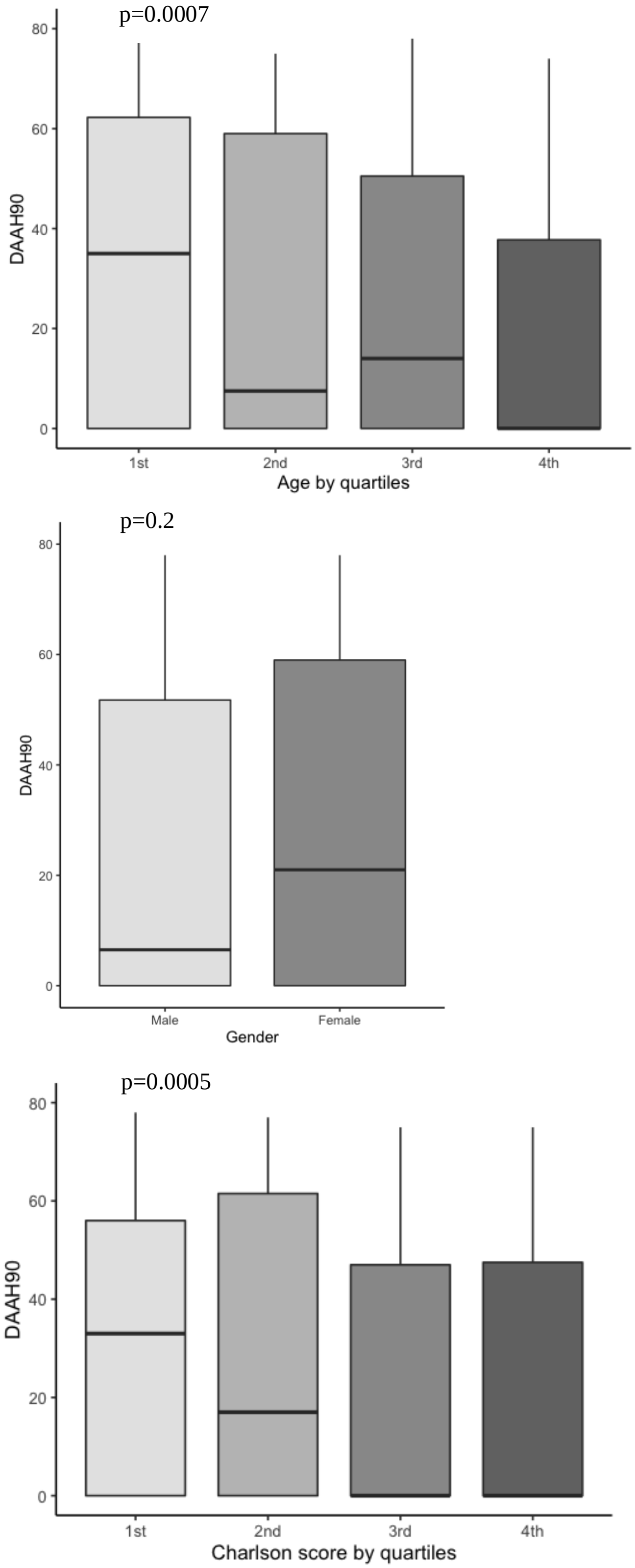

© 2023 Taran S et al. *JAMA Network Open*.

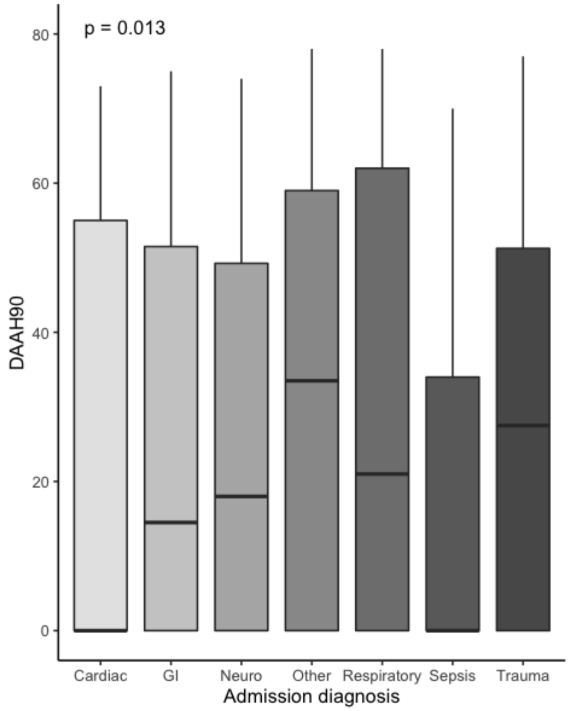

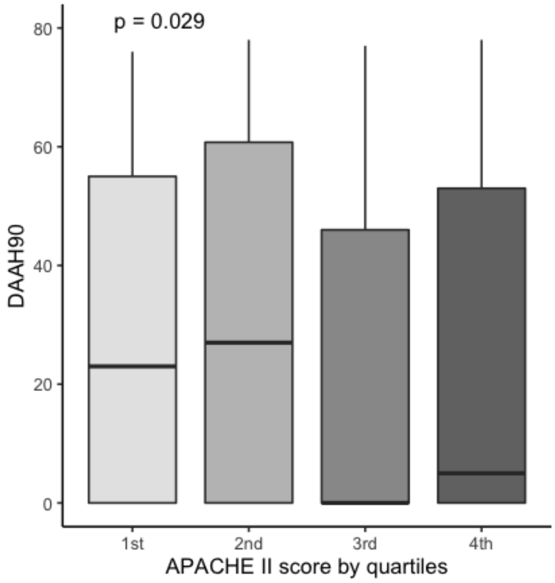

© 2023 Taran S et al. *JAMA Network Open*.

eTable 1. Association of Measures of Burden of Critical Illness With Tertiles of Days Alive and at Home at Day 90

	Unadjusted proportional odds ratio (odds ratio, 95% CI)	p-value	Adjusted proportional odds ratio* (odds ratio, 95% CI)	p-value
Duration of mechanical ventilation (days)	0.95 (0.94 – 0.97)	<0.0001	0.95 (0.93 – 0.96)	<0.0001
Intensive care unit length-of-stay among survivors (days)	0.93 (0.92 – 0.950	<0.0001	0.92 (0.91 – 0.94)	<0.0001
Ventilator-free days at day 28 (days)	1.13 (1.10 – 1.16)	<0.0001	1.14 (1.10 – 1.17)	<0.0001
Intensive care unit-free days at day 28 (days)	1.14 (1.11 – 1.17)	<0.0001	1.16 (1.12 – 1.20)	<0.0001
Tracheostomy	0.30 (0.21 – 0.42)	<0.0001	0.31 (0.21 – 0.45)	<0.0001
Renal replacement therapy	0.26 (0.17 – 0.41)	<0.0001	0.23 (0.14 – 0.38)	<0.0001

*Adjusted for age, sex, APACHE II score, and Charlson comorbidity index. Models were fitted using data from the entire baseline cohort.

© 2023 Taran S et al. *JAMA Network Open*.

eTable 2. Baseline Characteristics of the 292 Patients in the Follow-up Cohort

Characteristic	Median [IQR]			p-value
	$DAAH_{90}$ (1st tertile)	$DAAH_{90}$ (2nd tertile)	$DAAH_{90}$ (3rd tertile)	
Number of patients, n	98	97	97	
$DAAH_{90}$ (days), median [range]	0 [0–1]	30 [2–52]	65 [53–78]	
Age (years)	58.5 [49.5–69.8]	59.1 [47.9–66.1]	53.6 [42.0–62.1]	0.004
Female sex, n (%)	43 (43.9)	38 (39.2)	42 (43.3)	0.77
BMI (kg/m^2)	27.2 [25.6–31.6]	27.1 [24.4–31.6]	28.7 [24.7–34.8]	0.60
Education level, n (%)				0.99
Less than secondary	19 (19.4)	20 (20.6)	21 (21.6)	
Secondary/some post-secondary	33 (33.7)	33 (34.0)	33 (34.0)	
Post-secondary	46 (46.9)	44 (45.4)	43 (44.3)	
Prior Employment, n (%)				0.06
Part/full time work	45 (48.9)	47 (49.5)	48 (51.1)	
Retired	27 (29.3)	29 (30.5)	14 (14.9)	
Disability	9 (9.8)	9 (9.5)	10 (10.6)	
Other	11 (12.0)	10 (10.5)	22 (23.4)	
Income, n (%)				0.13
<$50,000	22 (23.9)	27 (28.4)	40 (42.6)	
$50,000−70,000	10 (10.9)	12 (12.6)	10 (10.6)	
>$70,000$	26 (28.3)	28 (29.5)	24 (25.5)	
Not known	34 (37.0)	28 (29.5)	20 (21.3)	
Charlson comorbidity index	1 [0–3]	1 [0–2]	1 [0–2]	0.48
Admission diagnosis category, n (%)				0.02
Cardiac	8 (8.2)	7 (7.2)	11 (11.3)	
GI	14 (14.3)	12 (12.4)	13 (13.4)	
Neuro	24 (24.5)	16 (16.5)	11 (11.3)	
Respiratory	18 (18.4)	29 (29.9)	36 (37.1)	
Sepsis	13 (13.3)	11 (11.3)	4 (4.1)	
Trauma	15 (15.3)	12 (12.4)	7 (7.2)	
Other	6 (6.1)	10 (10.3)	15 (15.5)	
APACHE II	22.5 [17–27]	21 [17–28]	22 [16–29]	0.79
Lowest PaO_2/FiO_2	114.3 [74–154]	101 [77–168]	103 [75–141]	0.64
Renal replacement therapy, n (%)	32 (32.7)	18 (18.6)	11 (11.3)	0.001
Tracheostomy, n (%)	66 (67.3)	43 (44.3)	21 (21.6)	<0.001
Duration of mechanical ventilation, days	24 [15–36]	19 [13–25]	11 [9–15]	<0.001
Ventilator-free days, days	3 [0–13]	9 [2–16]	17 [14, 19]	<0.001
ICU length-of-stay, days	29 [18–42]	22 [14–32]	13 [11–18]	<0.001
ICU-free days, days	0 [0–10]	6 [0–14]	15 [10–17]	<0.001
Index hospitalization length-of-stay, days	96 [66–152]	49 [40–65]	25 [20–31]	<0.001

© 2023 Taran S et al. *JAMA Network Open*.

Days in hospital for subsequent hospital admissions, days	21 [0–46]	19 [6–31]	0 [0–5.75]	<0.001
Days in long-term care	0 [0–0]	0 [0–0]	0 [0–0]	0.08
FIM score at day 7 after ICU discharge	42 [28–55]	51 [30–68]	93 [80–110]	<0.001
Total MRC	38 [24–47]	48 [42–54]	54 [49–58]	<0.001
Disability risk groups [a]				<0.001
Young, short LOS	0 (0.0)	3 (3.1)	5 (15.5)	
Mixed age, variable LOS	22 (22.4)	34 (35.1)	51 (52.6)	
Older, long LOS	45 (45.9)	37 (38.1)	28 (28.9)	
Oldest, long LOS	31 (31.6)	23 (23.7)	3 (3.1)	
Discharge disposition, n (%)				<0.001
Acute/Chronic Care	24 (25.3)	6 (6.2)	11 (11.3)	
Home	10 (10.5)	32 (33.0)	64 (66.0)	
Referring Hospital	16 (16.8)	15 (15.5)	12 (12.4)	
Rehabilitation	45 (47.4)	44 (45.4)	10 (10.3)	
Died in ICU	19 (100.0)	13 (100.0)	3 (100.0)	
Died in hospital	98 (100.0)	97 (100.0)	97 (100.0)	
One-year mortality, n (%)	11 (11.2)	5 (5.2)	1 (1.0)	0.009

[a] Defined in the original RECOVER study as follows: *young, short LOS (age < 42 yr and ICU LOS < 2 wk); mixed-age, variable LOS (age ≥ 42 yr and ICU LOS < 2 wk and age ≤ 45 yr and ICU LOS ≥ 2 wk); older, long LOS (age 46–66 and ICU LOS ≥ 2 wk); and oldest, long LOS (age >66 yr; ICU LOS ≥ 2 wk)).*

Abbreviations: APACHE, acute physiology and chronic health evaluation; BMI, body mass index; $DAAH_{90}$, days alive and at home at day 90; FIM, functional independence measure; GI, gastrointestinal; ICU, intensive care unit; MRC, medical research council; PaO_2/FiO_2, ratio of partial pressure of arterial oxygen to fraction of inspired oxygen

© 2023 Taran S et al. *JAMA Network Open*.

eFigure 3. Distribution of Days Alive and at Home at Day 90 ($DAAH_{90}$) According to Disability Risk Groups Defined in the RECOVER Study

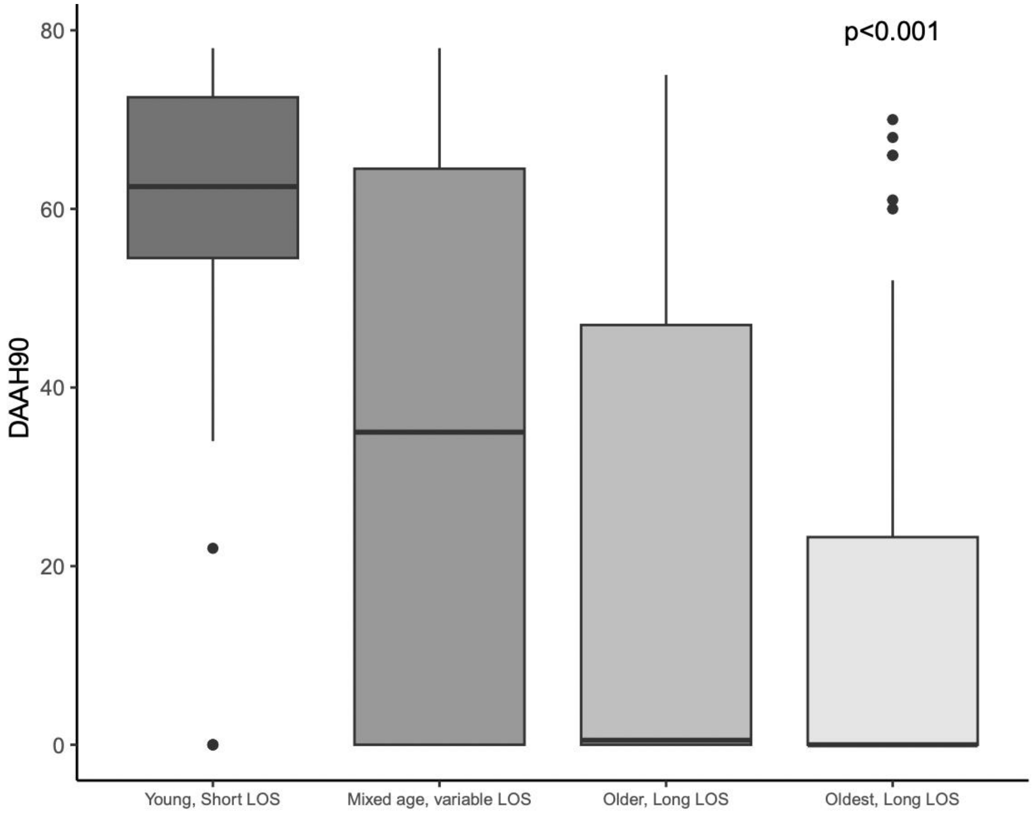

Disability risk groups were defined in the original study as follows: *young, short LOS (age < 42 yr and ICU LOS < 2 wk); mixed-age, variable LOS (age ≥ 42 yr and ICU LOS < 2 wk and age ≤ 45 yr and ICU LOS ≥ 2 wk); older, long LOS (age 46–66 and ICU LOS ≥ 2 wk); and oldest, long LOS (age >66 yr; ICU LOS ≥ 2 wk)).* Boxplots were created using data from the baseline cohort.

Abbreviations: $DAAH_{90}$, days alive and at home at day 90; LOS, length of stay

© 2023 Taran S et al. *JAMA Network Open*.

eTable 3. Adjusted Association of Composite Metrics With Functional Independence Measure Score at 1 Year

Metric	Estimate	95% CI	p-value
**$DAAH_{90}$**			
$DAAH_{90}$ (2nd)	12.5	5.0 – 20.0	**<0.01**
$DAAH_{90}$ (3rd)	22.4	14.8 – 30.0	**<0.0001**
Age	-0.15	-0.3 – 0.04	0.13
Female sex	-8.5	-14.7 – -2.2	<0.01
APACHE II score	0.3	-0.1 – 0.7	0.12
Charlson score	0.6	-1.4 – 2.5	0.57
**VFD_{28}**			
VFD_{28} (2nd)	5.6	-2.352 – 13.5	0.17
VFD_{28} (3rd)	6.0	-2.183 – 14.1	0.15
Age	-0.2	-0.438 – -0.034	0.02
Female sex	-8.9	-15.6 – -2.2	0.01
APACHE II score	0.4	-0.01 – 0.8	0.06
Charlson score	0.7	-1.4 – 2.8	0.52
**$ICU\text{-}FD_{28}$**			
$ICU\text{-}FD_{28}$ (2nd)	0.4	-7.6 – 8.4	0.93
$ICU\text{-}FD_{28}$ (3rd)	5.9	-2.1 – 13.8	0.15
Age	-0.2	-0.4 – -0.04	0.02
Female sex	-8.9	-15.6 – -2.1	0.01
APACHE II score	0.4	-0.02 – 0.8	0.06
Charlson score	0.7	-1.4 – 2.7	0.53

The adjusted associations between tertiles of each metric and FIM are quantified using linear regression. Data are reported for patients who survived to 3 months and who had FIM recorded at 1 year. Associations are reported against the first tertile (reference level) for each metric.

Abbreviations: APACHE, acute physiology and chronic health evaluation; $DAAH_{90}$ days alive and at home at day 90; FIM, functional independence measure; $ICU\text{-}FD_{28}$, intensive care unit-free days at day 28; VFD_{28}, ventilator-free days at day 28

© 2023 Taran S et al. *JAMA Network Open*.

eTable 4. Sensitivity Analysis Removing Long-Term Care Length of Stay at Day 90

Characteristic	Median (IQR)			Unadjusted p-value	Adjusted p-value*
	$DAAH_{90}$ (1st tertile)	$DAAH_{90}$ (2nd tertile)	$DAAH_{90}$ (3rd tertile)		
Number of patients with follow-up at 3 months, n	98	97	97		
$DAAH_{90}$ (days), median [range]	0 [0, 1]	30 [2, 52]	65 [53, 78]		
Functional independence measure					
3 months	78 [44–102]	116 [101–121]	121 [113–125]	<0.001	0.05
6 months	103 [66–116]	118.5 [110–123]	122 [115–125]	<0.001	0.06
12 months	104 [92–118]	121 [108–125]	123 [118–125]	<0.001	<0.001
6-minute walk test					
3 months	100 [0–240]	320 [220–452]	400 [300–496]	<0.001	<0.001
6 months	264 [100–348]	408 [294–490]	436 [294–522]	<0.001	<0.001
12 months	328 [151–417]	408 [254–514]	450 [372–545]	<0.001	<0.001
Medical research council score					
3 months	48 [33–54]	52 [48–58]	58 [51–60]	<0.001	<0.001
6 months	48 [44–58]	58 [48–60]	60 [54–60]	<0.001	<0.001
12 months	56 [48–60]	60 [52–60]	60 [58–60]	<0.001	<0.001
SF-36 Physical Component Score					
3 months	30 [22–38]	33 [27–41]	37 [31–47]	0.001	<0.001
6 months	30 [24–37]	37 [32–43]	40 [31–50]	<0.001	<0.001
12 months	33 [27–40]	40 [32–47]	41 [30–52]	0.001	<0.001
Impact Event Score					
3 months	15 [3–26]	12 [4–30]	18 [5–30]	0.61	0.91
6 months	12 [4–28]	15 [4–26]	13 [4–30]	0.83	0.61
12 months	14 [7–28]	11 [3–23]	10 [3–27]	0.27	0.19
Beck Depression Inventory					
3 months	12 [7–22]	9 [6–16]	9 [6–16]	0.26	0.10
6 months	11 [5–18]	8 [4–12]	10 [4–19]	0.17	0.11
12 months	10 [6–18]	7 [4–13]	8 [2–17]	0.20	0.20
One-year mortality, n (%)	10 (10.2)	6 (5.8)	1 (1.1)	0.03	0.02

Abbreviations: $DAAH_{90}$, days alive and at home at day 90; SF-36, Short Form-36

© 2023 Taran S et al. *JAMA Network Open*.

eTable 5. Cox Proportional Hazards Regression Model for 1-Year Mortality

	Adjusted hazard ratio (95% CI)	Adjusted p-value
$DAAH_{90}$ (2nd)	0.28 (0.12 – 0.64)	<0.01
$DAAH_{90}$ (3rd)	0.18 (0.07 – 0.43)	<0.001
Age	1.02 (1.00 – 1.05)	0.10
Female sex	1.37 (0.73 – 2.57)	0.33
APACHE II score	0.97 (0.93 – 1.02)	0.24
Charlson score	1.42 (1.20 – 1.68)	<0.001

The analysis was completed restricting to patients who were alive at day 90. Hazard ratios for $DAAH_{90}$ are reported against the first tertile (reference level).
Abbreviations: APACHE, acute physiology and chronic health evaluation; $DAAH_{90}$, days alive and at home at day 90

© 2023 Taran S et al. *JAMA Network Open*.

eFigure 4. Survival at 12 Months Among Survivors at Day 90 According to Tertiles of Days Alive and at Home at Day 90 Removing Long-Term Care Length of Stay

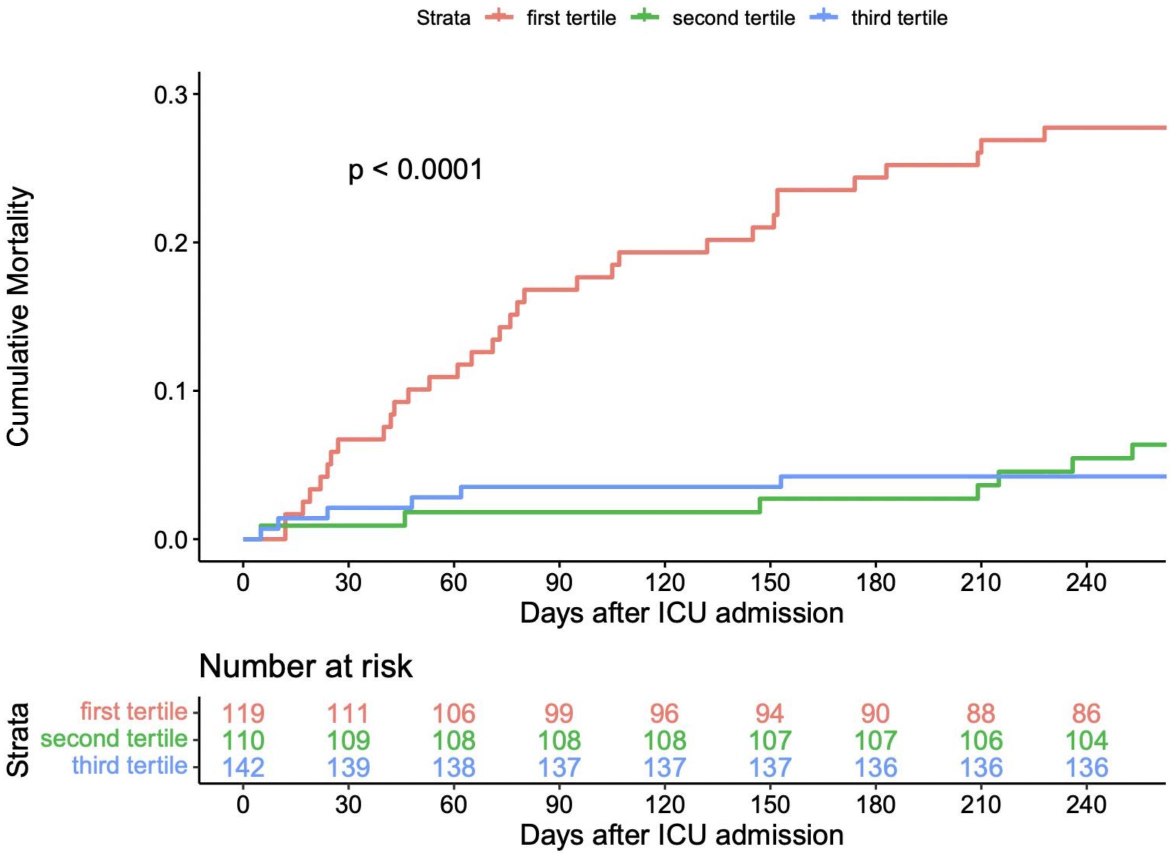

© 2023 Taran S et al. *JAMA Network Open*.

eFigure 5. Association of Deciles of Days Alive and at Home at Day 90 (DAAH$_{90}$) With Functional Outcomes at 3 Months

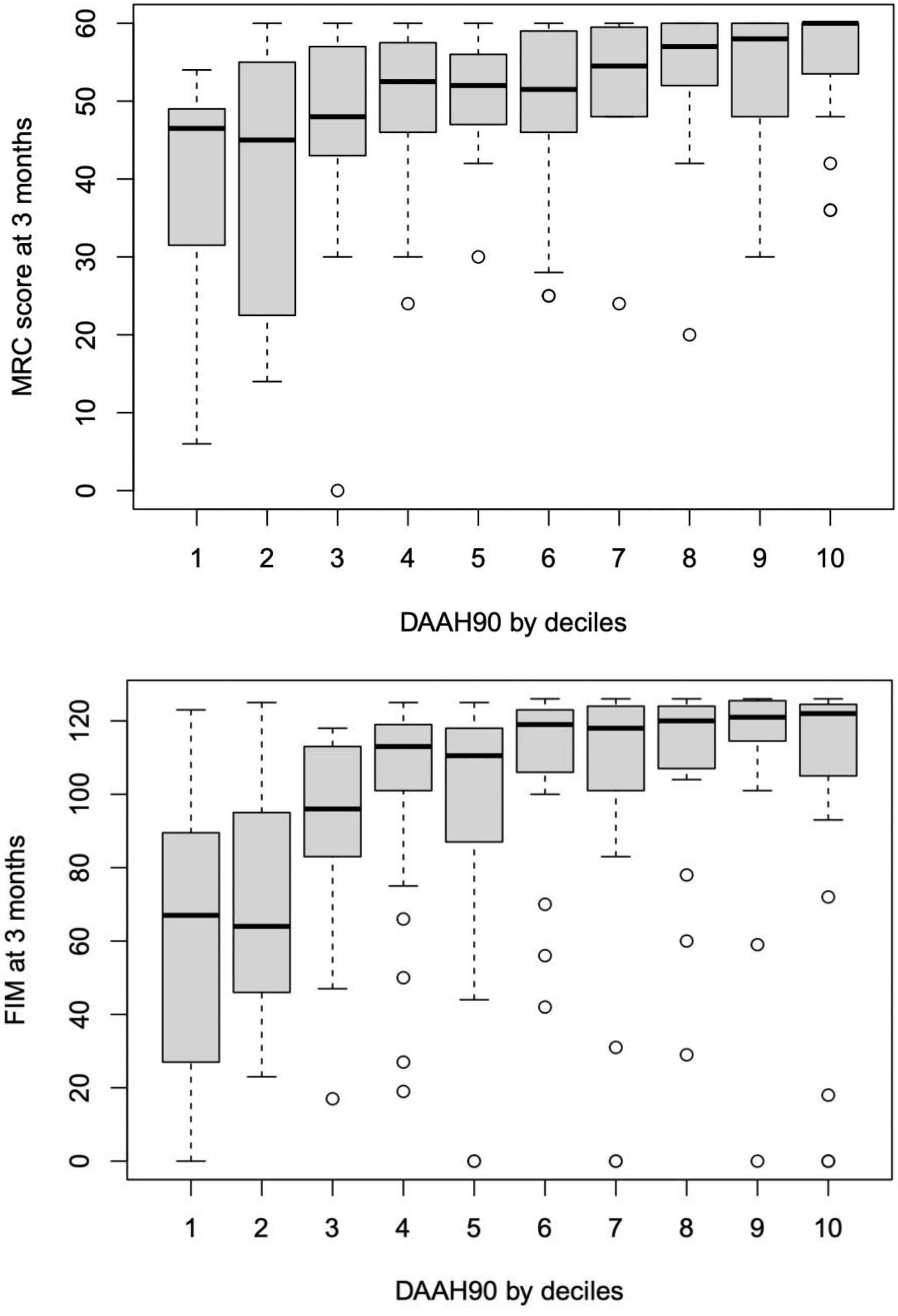

© 2023 Taran S et al. *JAMA Network Open*.

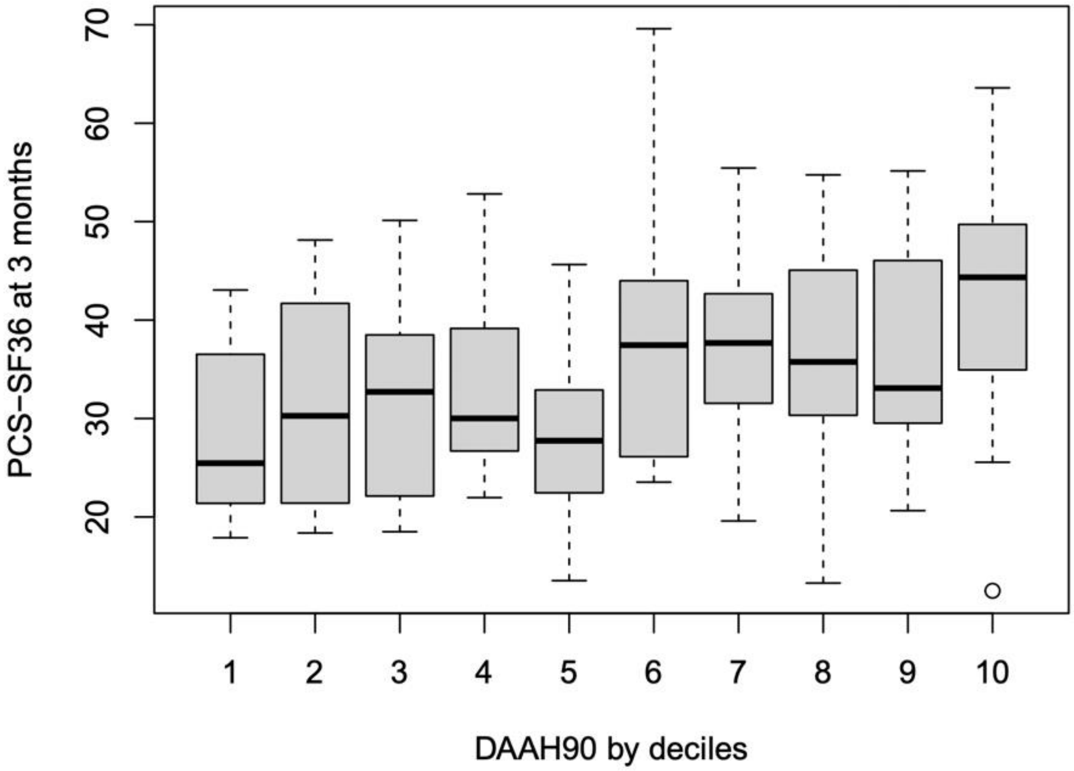

Values for each decile of $DAAH_{90}$ are as follows: 1st decile, 0; 2nd decile, 0; 3rd decile, 0; 4th decile, 0-16; 5th decile, 16-30; 6th decile, 31-46; 7th decile, 46-55; 8th decile, 56-63; 9th decile, 63-70; 10th decile, 70-78

Abbreviations: $DAAH_{90}$, days alive and at home at day 90; FIM, functional independence measure; MRC, medical research council; PCS-SF36, physical component score of the Short Form-36

© 2023 Taran S et al. *JAMA Network Open*.

eFigure 6. Mortality at 1 Year in Survivors at Day 90 According to Deciles of Days Alive and at Home at Day 90 ($DAAH_{90}$)

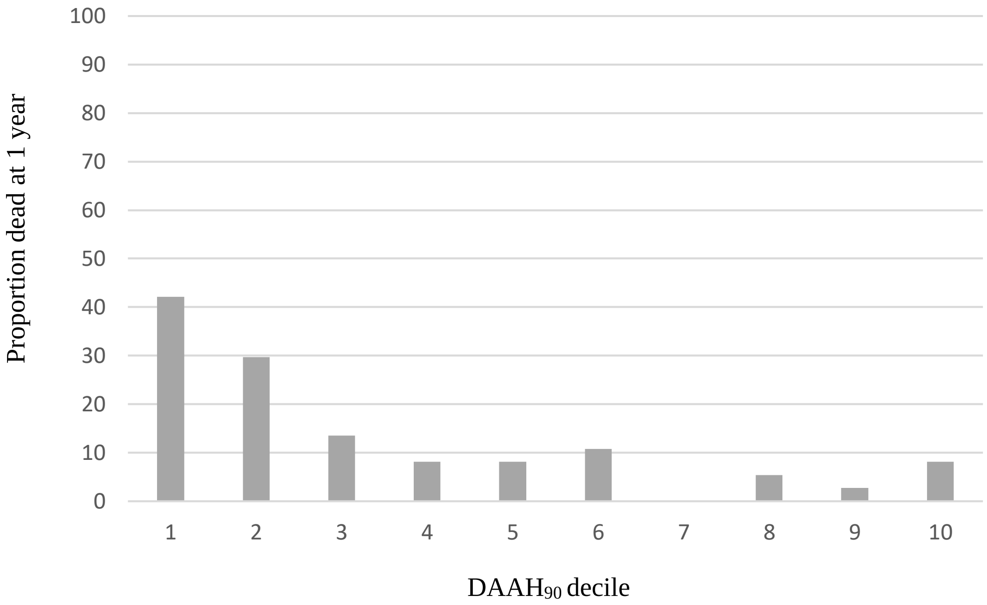

Values for each decile of $DAAH_{90}$ are as follows: 1st decile, 0; 2nd decile, 0; 3rd decile, 0; 4th decile, 0-16; 5th decile, 16-30; 6th decile, 31-46; 7th decile, 46-55; 8th decile, 56-63; 9th decile, 63-70; 10th decile, 70-78

Abbreviations: $DAAH_{90}$, days alive and at home at day 90

© 2023 Taran S et al. *JAMA Network Open*.
