# Supplementary material for: Association of Days Alive and at Home at Day 90 After Intensive Care Unit Admission With Long-term Survival and Functional Status Among Mechanically Ventilated Patients
Source: JAMA Netw Open. 2023 Mar 16;6(3):e233265. doi: 10.1001/jamanetworkopen.2023.3265 (PMC10020882; doi:10.1001/jamanetworkopen.2023.3265)
Supplement: Supplement 2. — Data Sharing Statement [file jamanetwopen-e233265-s002.pdf]

Data Sharing Statement

Taran. Association of Days Alive and at Home at Day 90 After Intensive Care Unit Admission With Long-term Survival and Functional Status Among Mechanically Ventilated Patients. *JAMA Netw Open*. Published March 16, 2023. doi:10.1001/jamanetworkopen.2023.3265

Data
Data available: No
